# *Staphylococcus sciuri* Exfoliative Toxin C (ExhC) is a Necrosis-Inducer for Mammalian Cells

**DOI:** 10.1371/journal.pone.0023145

**Published:** 2011-07-29

**Authors:** Haihua Li, Yongqiang Wang, Lin Ding, Shijun J. Zheng

**Affiliations:** State Key Laboratory of Agrobiotechnology, Key Laboratory of Animal Epidemiology and Zoonosis, and Ministry of Agriculture, College of Veterinary Medicine, China Agricultural University, Beijing, China; Columbia University, United States of America

## Abstract

*Staphylococcus sciuri* (*S. sciuri*) is a rare pathogen in humans, but it can cause a wide array of human infections. Recently a *S. sciuri* isolate (HBXX06) was reported to cause fatal exudative epidermitis (EE) in piglets and thus considered as a potential zoonotic agent. To investigate the pathogenicity of this bacterium, we cloned *exfoliative toxin C* (*ExhC*), a major toxin of the *S. sciuri* isolate and performed functional analysis of the recombinant ExhC-his (rExhC) protein using *in vitro* cell cultures and newborn mice as models. We found that rExhC could induce necrosis in multiple cell lines and peritoneal macrophages as well as skin lesions in newborn mice, and that the rExhC-induced necrosis in cells or skin lesions in newborn mice could be completely abolished if amino acids 79-128 of rExhC were deleted or blocked with a monoclonal antibody (3E4), indicating aa 79-128 portion as an essential necrosis-inducing domain. This information contributes to further understandings of the mechanisms underlying *S*. *sciuri* infection.

## Introduction

*Staphylococcus sciuri* (*S. sciuri*) is a rare pathogen in humans, but it can cause a wide array of human infections, such as endocarditis [1], peritonitis [2], septic shock [3], urinary tract infection [4], pelvic inflammatory disease [5] and wound infections [6], [7]. Recently a *S. Sciuri* isolate (HBXX06) that carry *exfoliative toxin C* (*ExhC*) was reported to cause an outbreak of fatal exudative epidermitis (EE) in piglets [8]. EE in pigs, also known as greasy pig disease, is an acute and communicable skin disease characterized by appearance of generalized exfoliation of epidermis accompanied by extensive exudation and crust formation [8]–[11]. Exfoliative toxins, major toxins produced by the causative agents, are responsible for the characteristic skin lesions [8], [9], [11]. Staphylococcal scalded skin syndrome (SSSS) in humans caused by *Staphylococcus aureus* (*S. aureus*) strains shared similar clinical signs and histopathology with EE in pigs, exhibiting blister formation and exfoliation of the skin caused by the skin splitting at the granular layer of the epidermis [12]–[16]. Thus, EE may be used as a disease model to elucidate the mechanisms of *Staphylococcus* infections in humans.

Exfoliative toxins are critical virulence factors responsible for the pathogenesis of EE in pigs. Currently, at least six exfoliative toxins, ExhA through D, ShetA and ShetB, have been identified and purified from different strains [10], [11], [17], and their existence is related to the species of *Staphylococci* [11], [18], [19]. These toxins have been characterized as proteins of approximately 27 kDa or 30 kDa [14], [20], [21]. Target molecules for exfoliative toxins ExhA-D in swine have been identified as the extracellular domains of desmoglein (Dsg) 1, a cell-cell adhesion molecule in desmosomes [22]. In addition, ExhA and ExhC are able to cleave mouse Dsg 1α and 1β [9], which may allow the use of mice as animal models for exploring the biological activities of Staphylococcal exfoliative toxins. Previous reports showed that exfoliative toxins from *S. hyicus* could cause rounding effects in mammalian cells and skin lesions in newborn mice [9], [23]. However, the exact mechanisms underlying the cell death caused by exfoliative toxins are not clear.

In this study, we showed that recombinant ExhC (rExhC) caused necrosis in multiple cell lines and peritoneal macrophages as well as skin lesions in newborn mice, and that the rExhC-induced necrosis in cells or skin lesions in mice could be completely abolished if amino acids 79-128 of rExhC were deleted or blocked with a monoclonal antibody (3E4), indicating the amino acids 79-128 portion of ExhC as an essential necrosis-inducing domain.

## Results

### Recombinant ExhC-his proteins caused skin lesions in newborn mice

**Figure 1:**
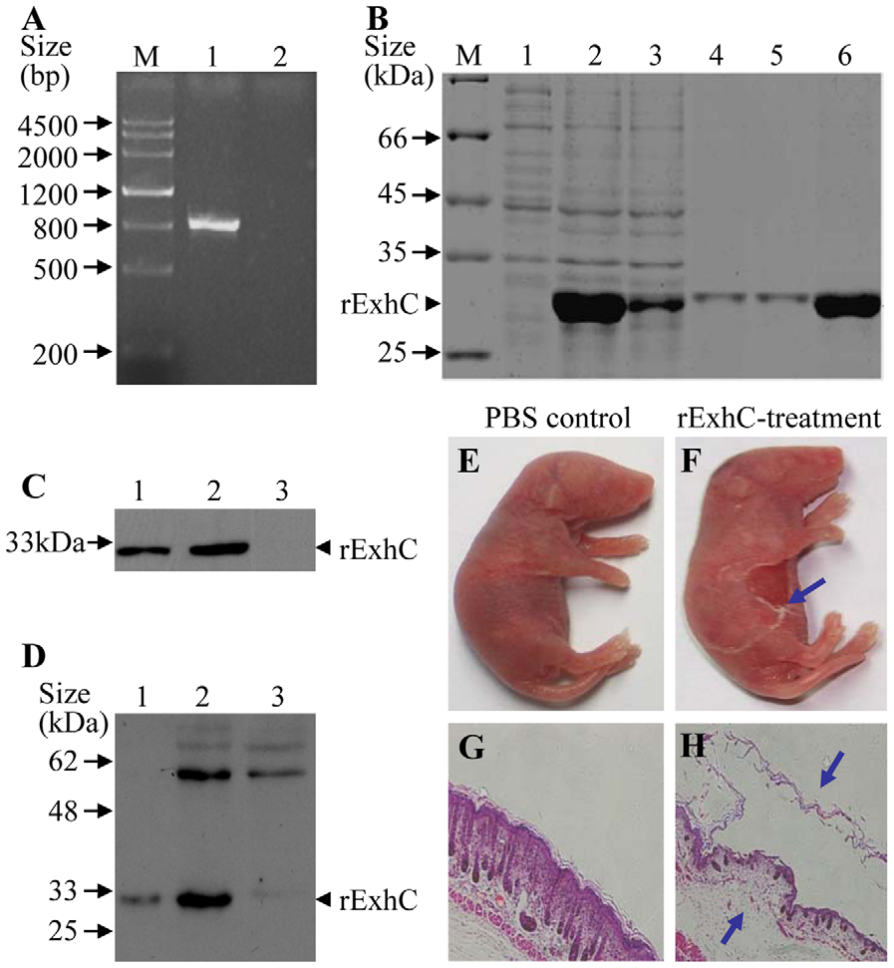
Recombinant ExhC-his proteins caused skin lesions in newborn mice. A. *ExhC* was amplified from genomic DNA of *S. sciuri* isolate (Lane 1) with distilled water as a control (Lane 2) using specific primers. M stands for DNA Marker. B. SDS-PAGE analysis of the purified rExhC. Lane 1 was loaded with cell extracts of empty vector, lane 2 with cell extracts of rExhC, lane 3 with flow-through buffer solution, lanes 4 & 5 with wash buffer, and lane 6 with purified rExhC. M represents standard protein markers. C and D. The expression of rExhC was examined by Western blot using anti-his McAb (C) and polyclonal antibodies against *S. sciuri* (D). Lane 1 was loaded with purified rExhC, lane 2 with cell extracts of rExhC, and Lane 3 with empty-vector transformed cell extracts. E–H. Recombinant ExhC-his proteins cause exfoliation of skins in newborn mice. E & F. newborn mice were injected subcutaneously with PBS as controls (E) or rExhC (F). Six h later, the gross lesions were examined. G & H. Histological examination of skin lesions in controls (G) or rExhC-injected mice (H). Arrows in F and H indicates the lesions in the skin of mice. Results are representative of two independent experiments with the similar results. Original amplification is ×200.

In our previous report, we showed that *ExhC* was the only exfoliative toxin in the genome of pathogenic *S. sciuri* isolate (HBXX06) [8]. To explore the biological activity of ExhC, we amplified the *ExhC* (837 bp) from the genome of *S. sciuri* isolate (HBXX06) by PCR using specific primers (Figure 1A). Sequencing analysis of the PCR product indicated that the *S. sciuri ExhC* (GenBank ID: JF755400) was identical to that of *S. hyicus* (GenBank ID: AF515455) [10]. We made a pET28a(+)-ExhC expression construct, and expressed the rExhC protein using *E. coli* expression system. The rExhC protein was purified with Ni-NTA columns and examined by SDS-PAGE and Western Blot. As shown in Figure 1B, the rExhC was successfully expressed and purified as examined by SDS-PAGE. In addition, the rExhC could be detected with anti-his tag monoclonal antibody (Figure 1C) or rabbit anti-*S. sciuri* isolate (HBXX06) serum (Figure 1D), suggesting that ExhC is an immunogenic component of the *S. sciuri* isolate.

Since newborn mice are sensitive to ExhC [9], we used newborn mice as a model to examine the biological activity of rExhC. As shown in Fig. 1E & F, newborn mice displayed blistering and exfoliation of the skin 6 hours after subcutaneous injection with 500 µg of purified rExhC while no clinical signs were observed in controls. Consistently, histological examination also showed that the exfoliated epidermis and necrosis in the dermis only existed in the skin tissue of rExhC-treated mice but not in controls (Figures 1G & 1H). These data suggest that the rExhC is a potent toxin causing tissue damages and can be used to elucidate the functions of ExhC.

### rExhC induced necrosis in cells

**Figure 2:**
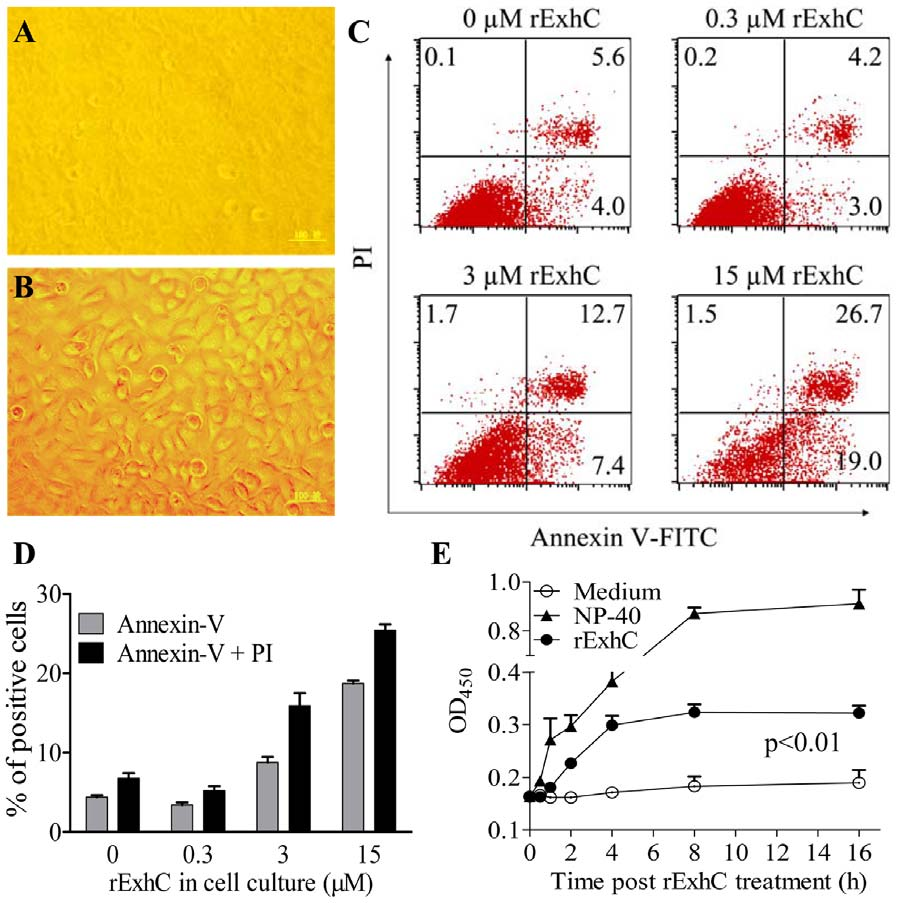
rExhC induced necrosis in cells. A and B. BHK-21 cells were cultured with (A) or without (B) rExhC for 24 h and observed with a microscope. C and D. BHK-21 cells were treated with indicated amounts of rExhC for 24 h before stained with FITC-labeled annexin-V and PI, and were analyzed by flow cytometry. The significance of the differences between rExhC-treated and control cells in terms of positive cells rate was performed by *ANOVA* (*p*<0.001). E. BHK-21 cells were cultured with 15 µM rExhC or 0.1% NP-40 as a positive control or medium only as a negative control at the indicated times and the DNA contents in culture supernatants were determined using a DNA detection ELISA kit. Results are from one representative of three independent experiments and presented as means ± SEM. Statistical analysis was performed using *ANOVA*.

To analyze the functions of rExhC, we cultured BHK-21 cells with or without rExhC. We found that cells treated with rExhC underwent intensive cell death (Figure 2A) whereas controls grew well (Figure 2B), and that the rExhC-induced cell death was dose-dependent as examined by flow-cytometry using Annexin-V and PI staining (Figures 2C & 2D). To determine if rExhC could induce cell death in other cell types, L-929, RAW264.7 and B16 cells as well as mouse peritoneal macrophages were cultured with rExhC. Interestingly, all these cells were sensitive to rExhC-induced cell death (data not shown), which suggests that rExhC may induce cell death in both cell lines and primary cell culture. To analyze the rExhC-induced cell death, we examined apoptosis and necrosis by measuring DNA fragmentation, caspase cleavage and supernatant DNA contents in the cell culture after rExhC treatment. We found that neither fragmented DNA nor cleaved caspase 3 or 9 was detected in rExhC-treated cells (Figure S1A–C). In addition, rExhC-induced cell death was not abrogated by pancaspase inhibitor zVAD-fmk (Figure S1D). However, DNA contents in the supernatant of rExhC-treated cells were significantly greater than that of medium control (*p*<0.01) (Figure 2E). These data indicates that rExhC primarily induces necrosis rather than apoptosis in mammalian cells.

### A critical role of rExhC 79-128 aa portion in rExhC- induced necrosis in cells

**Figure 3:**
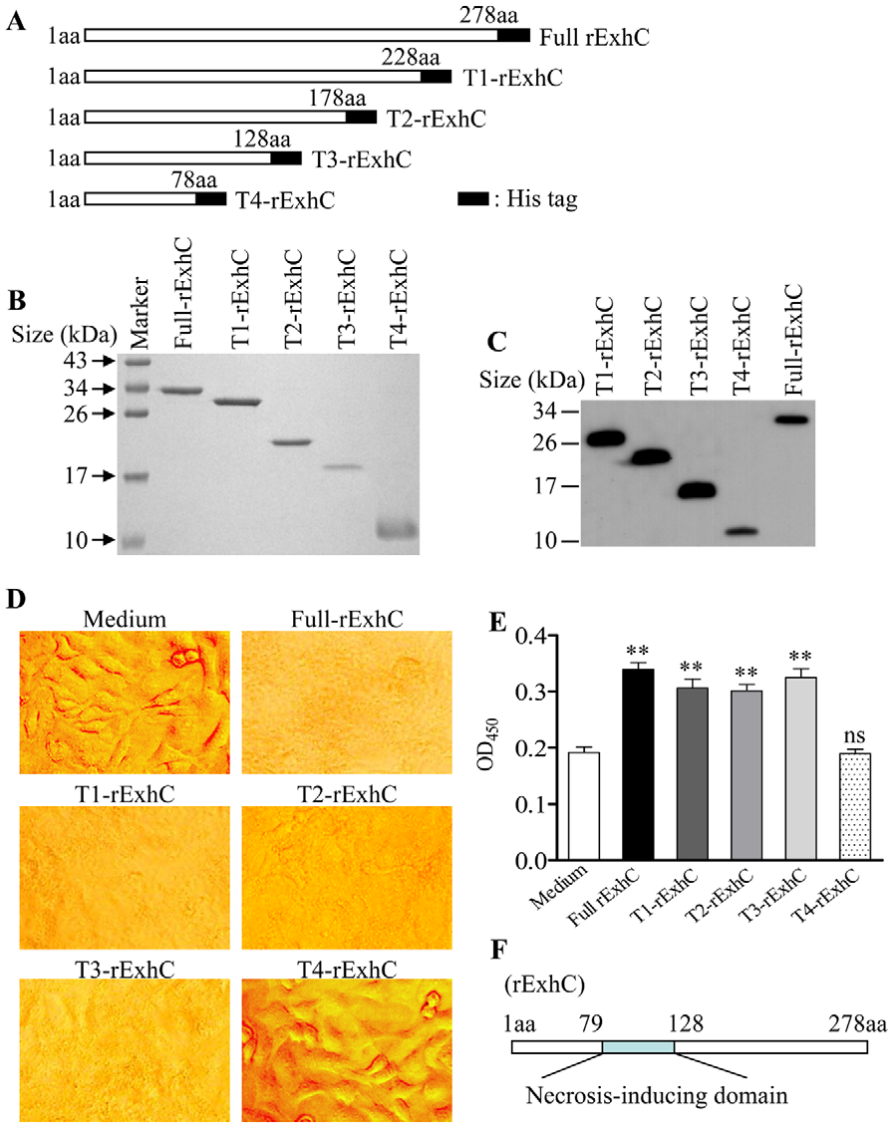
A critical role of rExhC 79-128 aa in the induction of necrosis in BHK-21 cells. A. Schematic diagrams showing the structure of full rExhC and truncated rExhCs (designated T1-rExhC, T2-rExhC, T3-rExhC and T4-rExhC). B. SDS-PAGE analysis confirms the purity of the truncated rExhCs. C. Western Blot analysis confirms the expression of truncated rExhCs using anti-his McAb. D and E. BHK-21 cells were cultured in medium only as controls, or cultured with 15 µM of rExhC, T1-rExhC, T2-rExhC, T3-rExhC and T4-rExhC respectively for 8 h, and observed with a microscope (D) before the DNA contents in culture supernatants were determined using a DNA detection ELISA kit (E). Arrows indicate necrotic cells. Results are from one representative of three independent experiments. Statistical analysis was performed using *ANOVA*. **, *p*<0.01; ns, not significant. F. Schematic diagrams showing the necrosis-inducing domain of rExhC.

Since rExhC induced necrosis in mammalian cells, it would be important to clarify the necrosis-inducing portion of this molecule. We made truncated pET-28(+)-rExhC constructs encoding 1-78, 1-128, 1-178 and 1-228 aa of rExhC respectively (Figure 3A) and expressed the rExhC proteins using *E. coli* expression system. The purified truncated rExhC proteins were examined by SDS-PAGE and Western Blot with an anti-his tag monoclonal antibody. As shown in Figures 3B & C, the truncated rExhC proteins were successfully expressed and purified. Then we cultured BHK-21 cells with rExhC, truncated rExhC (T1-rExhC, T2-rExhC, T3-rExhC or T4-rExhC) or medium controls and examined the necrotic effects of these truncated rExhC proteins on cells by microscopy and ELISA. Interestingly, we found that the rExhC, T1-rExhC, T2-rExhC and T3-rExhC proteins caused severe cell death whereas cells treated with T4-rExhC or medium controls grew well (Figure 3D). Consistent with this observation, the DNA contents in the supernatant of cell cultures with rExhC, T1-rExhC, T2-rExhC or T3-rExhC were significantly greater than that of the cells treated with T4-rExhC or medium controls (*p*<0.01) (Figure 3E), suggesting that the amino acids 79-128 portion of rExhC is a necrosis-inducing domain (Figure 3F).

### Inhibition of rExhC-induced necrosis by blocking amino acids 79-128 of rExhC with a monoclonal antibody

**Figure 4:**
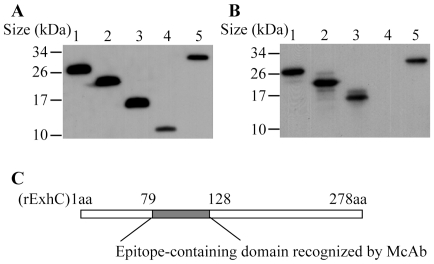
Anti-rExhC monoclonal antibody recognized aa 79-128 portion of rExhC. A and B. Western Blot analysis shows that rExhCs are recognized by anti-his monoclonal Ab (A) and monoclonal Ab 3E4-IgG1 (B). Lanes 1-5 were loaded with rExhC, T1-rExhC, T2-rExhC, T3-rExhC and T4-rExhC respectively. C. Schematic diagrams showing the epitope-containing domain recognized by monoclonal Ab 3E4-IgG1.

**Figure 5:**
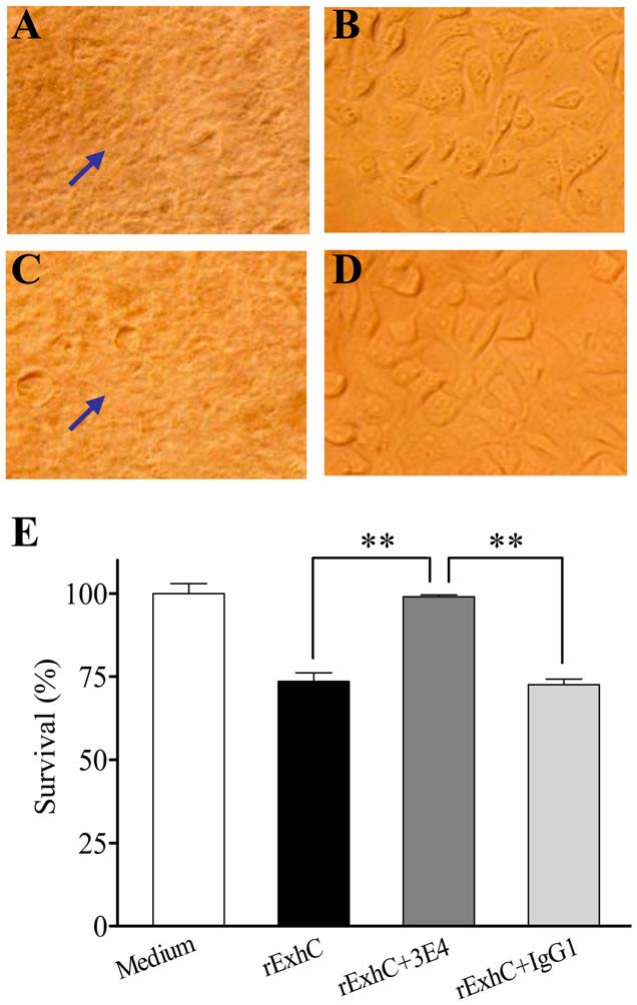
rExhC-induced necrosis was inhibited by blocking aa 79-128 portion of rExhC with a monoclonal antibody. BHK-21 cells were cultured with 15 µM rExhC (A) and also in the presence of 15 µM 3E4-Ab (B), 15 µM isotype IgG1 (C) or 15 µM 3E4-Ab only (D) as controls. Morphological changes were observed with a microscope 6 h post treatment. Arrows indicate necrotic cells. Twenty-four hours later, the cell viability was determined trypan blue dye exclusion assay (E). The significance of the differences between rExhC+3E4-treated and rExhC-treated cells in terms of survival rate was performed by *ANOVA* (*p*<0.01). Results are representative of three independent experiments with the similar results. **, *p*<0.01.

Since rExhC induced necrosis in cells via amino acids 79-128 domain, we hypothesized that aa 79-128 of rExhC might contain epitopes, and that the antibody raised against aa 79-128 domain would inhibit rExhC-induced necrosis. To test this hypothesis, we developed monoclonal antibodies and examined the neutralizing effects of these antibodies on rExhC-induced cell death. As shown in Figure 4A, all the truncated rExhC proteins were expressed with *E. coli* expression system as examined by Western Blot using anti-his monoclonal antibody. Interestingly, we found that one monoclonal antibody (clone #: 3E4-IgG1) recognized only those truncated rExhC proteins containing aa 79-128 of rExhC as demonstrated by Western Blot (Figure 4B), indicating that aa 79-128 of rExhC contained an epitope that could be recognized by this monoclonal antibody (Figure 4C). Next, we cultured BHK-21 cells with rExhC in the presence of the monoclonal antibody (3E4) or IgG1 controls. Strikingly, the rExhC-induced cell death could be effectively inhibited by the monoclonal antibody (3E4) (Figures 5A & B) but not by IgG1 control (Figure 5C) while the 3E4 antibody alone did not affect cell growth (Figure 5D). In addition, rExhC-treated cells survived much better after treatment with the 3E4 antibody than those treated with IgG1 controls (*p*<0.01) (Figure 5E). These data suggest that monoclonal antibody 3E4 can effectively inhibit rExhC-induced necrosis in cells via blocking aa 79-128 of rExhC.

### Inhibition of rExhC-induced skin lesions in newborn mice by a monoclonal antibody

**Figure 6:**
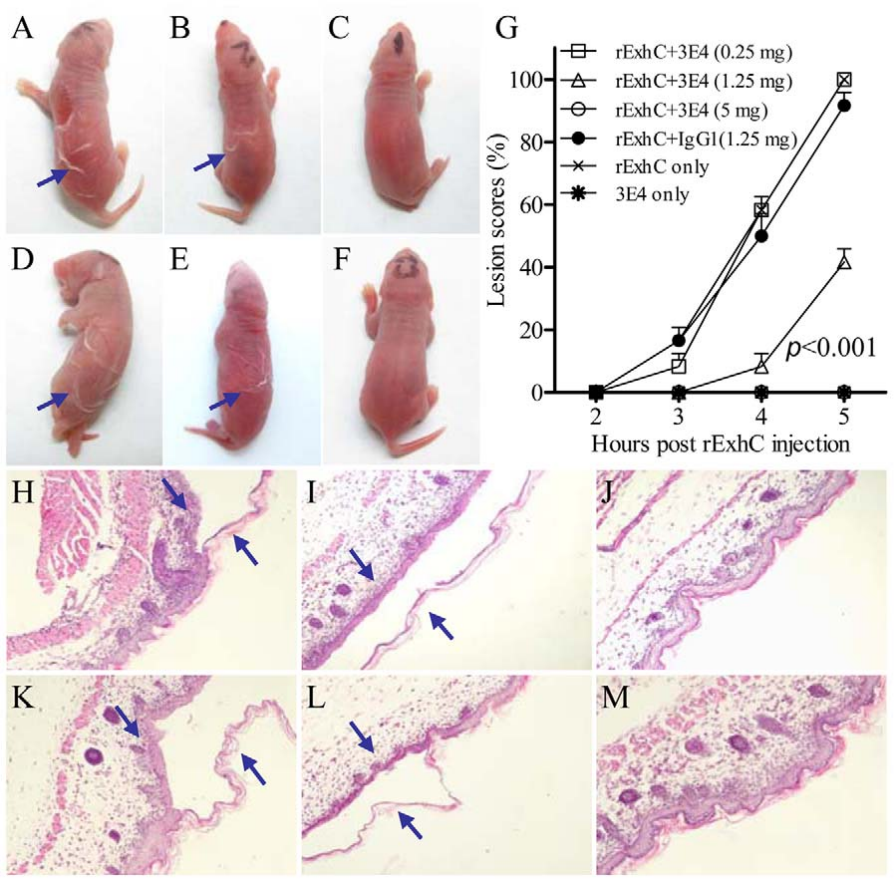
rExhC-induced skin lesions in newborn mice were inhibited by a monoclonal antibody. A–E. Newborn mice were treated with 0.25 mg (A), 1.25 mg (B) and 5 mg (C) of 3E4-Ab respectively, or with IgG1 (D)/PBS (E) as controls 1 h before subcutaneously injected with rExhC. Macroscopical skin lesions were observed 5 h post rExhC treatment. F. Mice were treated with 3E4-Ab only as a control. G. Skin lesions were recorded at the indicated time points after rExhC treatment in the presence of 0.25, 1.25 and 5 mg of 3E4-Ab respectively. rExhC+IgG1 (1.25 mg) was used as controls. The significance of the differences between rExhC+3E4-treated mice and rExhC+IgG1-controls in lesions was performed by *ANOVA* (*p*<0.001). H-M. Histological examination of skin tissues that were collected 5 h post rExhC injections. Mice were treated with 0.25 mg (H), 1.25 mg (I) and 5 mg (J) of 3E4-Ab respectively, or with IgG1 (K) as controls before injection with rExhC. Mice were also treated with rExhC only (L) or 3E4-Ab only (M) as controls. Original amplification is ×200. Results are representative of two independent experiments.

Since the monoclonal antibody (3E4) inhibited rExhC-induced necrosis in cells, it would be tempting to investigate whether the toxic effect of rExhC on the skin of newborn mice could also be inhibited by 3E4-Ab. We treated newborn mice with different doses of 3E4-Ab or IgG1 control via intraperitoneal injection one hour before the mice were subcutaneously injected with rExhC. Five hours after rExhC injection, low-dose 3E4-Ab-treated mice (Figure 6A), IgG1 (Figure 6D) and PBS (Figure 6E) controls developed severe skin lesions. In contrast, mice treated with a medium dose or a high dose of 3E4-Ab displayed slight or no skin lesions after rExhC treatment (Figures 6B & C), which indicates a protective role of 3E4-Ab in rExhC-induced skin lesions. Meanwhile, the mice treated with 3E4-Ab only looked healthy (Figure 6F), which suggests that the highly purified 3E4 had no visible side-effects.

To quantitatively analyze the toxic effects of rExhC, the scores of rExhC-induced skin lesions were recorded according to the size of skin lesion areas as described in Experimental procedures. Interestingly, mice injected with 3E4-Ab at a dose greater than 1.25 mg/mouse could be significantly protected from the rExhC-induced skin lesions as compared to the control or low-dose 3E4-Ab treatment groups (*p*<0.001) (Figure 6G), which suggests that the monoclonal antibody 3E4 provides protections against rExhC-induced skin lesions in mice in a dose-dependent manner. In addition, histological examination of the skin samples demonstrated that the mice pre-treated with a low- or a medium-dose of 3E4-Ab or IgG1 or PBS in advance displayed characteristic features of skin splitting at the granular layer after ExhC treatment (Figures 6H, 6I, 6K and 6L), but the mice pre-treated with a high dose of 3E4-Ab (Figure 6J) did not show any detectable lesions as compared to the control (Figure 6M). These *in vivo* data demonstrate that the rExhC-induced skin lesions in newborn mice can be effectively inhibited by a monoclonal antibody.

## Discussion

*S. sciuri* is widely distributed in nature, and strains can be easily isolated from a variety of animals and products of animal origin [24], [25] as well as from humans [26], [27]. These bacteria are normally nonpathogenic, but occasionally cause diseases in animals and humans [1]–[7]. It has been reported that some pathogenic strains of *S. sciuri* are responsible for mastitis in ruminants such as goats [28] and cows [29], suggesting that some members of *S. sciuri* are potentially pathogenic. In our previous study, we reported that a *S. sciuri* isolate (HBXX06) was highly pathogenic to piglets and it harbored ExhC as a major toxin [8]. In this report, we show that the rExhC induces necrosis on cell lines (BHK-21 cells, L-929, RAW264.7 and B16) and mouse peritoneal macrophages, and also cause the skin lesions in newborn mice.

Exfoliative toxins are critical virulence factors for causing EE in pigs. From *S. hyicus*, a commonly-seen causative agent for EE, four exfoliative toxins (ExhA-D) have been characterized [10]. Although certain strain of *S. hyicus* may have more than one exfoliative toxin [30], ExhC is the only exfoliative toxin produced by the *S. sciuri* isolate (HBXX06) [8]. Our data indicate that the *ExhC* from *S. sciuri* (HBXX06) is identical to that of *S. hyicus* in GenBank (AF515455). It was reported that the exfoliative toxins from *S. hyicus* and *S. aureus* are highly close to those found among the same species as within each species, leading to speculations that horizontal gene transfer may occur among species of *Staphylococci* [10]. Alternatively, it was proposed that “pathogenicity island” encoding staphylococcal virulence factors might be acquired by non-virulent strains by lysogenization [31]. Therefore, it was likely that *S. sciuri* isolate (HBXX06) acquired *ExhC* via horizontal gene transfer from the other *Exh*-carrying S*taphylococci*, such as *S. hyicus*. More efforts are required to investigate the mechanisms underlying the transmission of virulence factors among strains of *staphylococci*.

The present study was primarily focused on the biological activities of ExhC. Our results indicate that the purified rExhC protein is biologically active, which is consistent with the previous observation that ExhA and ExhC could cleave mouse Dsg 1α and 1β [9]. Interestingly, we found that neither cleavage of caspases nor DNA fragmentation was detected in rExhC-treated cells. Instead, a large amount of DNA was released from the rExhC-treated cells. Thus, rExhC cause necrosis rather than apoptosis in mammalian cells.

Our data indicate that aa 79-128 portion of rExhC determines the toxic effects of rExhC because rExhC-induced cell death in culture cells or the skin lesions in mice can be inhibited if the aa 79-128 portion of rExhC is deleted or blocked with 3E4-Ab. In comparison to those exfoliative toxins produced by other *Staphylococcus* subspecies such as *S. hyicus* ExhA (GenBank ID: AAN32970), ExhB (GenBank ID: BAA99411), ExhC (GenBank ID: AAN32972) and ExhD (GenBank ID: AAN32973), *S. aureus* ETA (GenBank ID: NP-510960), ETB (GenBank ID: NP-478350) and ETD (GenBank ID: BAC22944), *S. chromogenes* ExhB (GenBank ID: AAV98626), and *S. pseudintermedius* ExpB (GenBank ID: BAJ23893), *S. scuiri* ExhC contains 33 (11.9%) conservative aa sites while ten of them are located between 79-128 aa (20%), which indicates that the 79-128 aa portion is more conservative than the rest of the molecule. Interestingly, we found that mutant ExhC with a point mutation in H107, a conservative aa of ExhC, failed to cause skin lesions in newborn mice but could still induce necrosis in culture cells (data not shown), suggesting that the essential amino acids for cell necrosis and skin lesions might be different. More efforts are required to elucidate the discrepancy between ExhC-induced cell necrosis and skin lesions.

It was reported that a monoclonal antibody against exfoliative toxin from *S. hyicus* could not effectively neutralize the toxins from *S. hyicus* [14]. In our study, we developed several clones of monoclonal antibodies against rExhC, however only 3E4-Ab that recognized aa 79-128 domain of rExhC could effectively inhibit the rExhC-induced necrosis in culture cells or skin lesions in newborn mice. These results suggest that the aa 79-128 portion of rExhC acts as a critical domain responsible for inducing cell death, and that the ExhC may have multiple epitopes with variable functional domains. No doubt, further characterization of ExhC will help to elucidate the mechanisms of Staphylococcal scalded skin syndrome (SSSS) in humans because SSSS shares similar clinical signs and histopathology with EE in pigs.

In summary, we found that ExhC induced necrosis in mammalian cells and skin lesions in newborn mice, and that these toxic effects could be completely abolished if the aa 79-128 portion of rExhC was deleted or blocked with a monoclonal antibody (3E4), indicating the aa 79-128 portion as an essential necrosis-inducing domain. This information contributes to further understandings of the mechanisms underlying *S*. *sciuri* infection.

## Materials and Methods

### Mice

Eight-week-old inbred BALB/c mice were purchased from Vital River Lab Animal Technology Company (Beijing, China). All mice were housed in our animal care facility with food and water ad libitum for at least 3 days before mating. The newborn mice (less than 24 h) were used to determine the activity of rExhC and the protection of monoclonal antibody against ExhC.

### Ethics statement

All procedures were approved by the Animal Care and Use Committee of China Agricultural University (Approval IDs: XXMB-2007-03-01-1 and XXMBB-2007-03-15-1) and used in accordance with regulations and guidelines of this committee.

### Bacterial strains, cells and culture conditions

The *S. sciuri* strain (HBXX06) was originally isolated from the cardiac fluid of a diseased piglet with EE [8] and saved in our laboratory. The bacterium was grown in brain heart infusion medium (BHI) for extracting genomic DNA. *E. coli* DH5α (Tiangen Biotech) was grown in Lubia-Bertani (LB) medium with ampicillin (100 µg ml^−1^) for the preparation of plasmids. *E. coli* BL21 (DE3) (Tiangen Biotech) was grown in LB medium with kanamycin (50 µg ml^−1^) for the expression of rExhC. All strains were grown at 37°C unless otherwise specified. BHK-21 (baby hamster kidney cell line), L-929 (mouse fibroblast cell line), RAW264.7 (mouse macrophage cell line) and B16 (mouse melanoma cell line) cells as well as mouse peritoneal monocytes were used for functional analysis of rExhC, the cells were grown at 37°C with 5% CO_2_ in complete Dulbecco's modified Eagle medium (DMEM) (GIBCO) supplemented with 10% Fetal Bovine Serum (HyClone), 1% nonessential amino acids (GIBCO), and 200 U ml^−1^ penicillin and streptomycin.

### DNA manipulation

Genomic DNA was extracted from *S. sciuri* (HBXX06) using TIANamp Bacteria DNA kit (Tiangen Biotech). Plasmid DNA was prepared using TIANprep Mini Plasmid kit (Tiangen Biotech). Restriction enzymes and T4 DNA ligase were purchased from Takara (Japan). All enzymatic reactions were carried out according to the manufacture's instructions. DNA sequencing was performed by SinoGenoMax Co. (China). All new data have been deposited in GenBank (GenBank accession ID: JF755400)

### Construction of cloning and expression plasmids with ExhC

The *ExhC* gene was amplified from *S. sciuri* genomic DNA using the forward primers 5′- CCATGGCTATGCATTCAAAACTATTAAGTAAAT and the reverse primer 5′- GCGGCCGCTTTAATTAATTGTTTGAGATCTCTAATGAG with *Nco* Ι and *Not* Ι sites as underlined. PCR was performed with a program containing an initial step at 94°C for 4 min followed by 30 cycles for the amplification of *ExhC*, each cycle consisting of 94°C for 30 s, 50°C for 30 s, and 72°C for 60 s. The PCR products were purified before inserted into cloning plasmid pMD19-T simple vector (Takara), and the resulting plasmid, pMD19-T-ExhC, was used to transform *E. coli* DH5α. Transformants were grown on LB agar plates with ampicillin (100 µg ml^−1^) at 37°C and the colonies were screened by PCR and DNA sequencing analysis. The pMD19-T-ExhC and pET28a(+) (Novagen) empty plasmids were digested with *Nco* Ι and *Not* Ι, respectively. The Linearized *ExhC* and pET28a(+) were ligated with T4 DNA ligase (Takara) before sequencing analysis.

### Expression and purification of rExhC

*E. coli* BL21 (DE3) competent cells were transformed with the fusion constructs or empty plasmids and grown overnight at 37°C in LB medium with kanamycin (50 µg ml^∼1^). Transformants were grown to OD_600_ of 0.5-0.6 before supplemented with 0.5 mM IPTG and subsequently cultured at 16°C for 12 h. Bacterial cells were centrifuged at 6000 *g* for 5 min and frozen at −20°C till use. Recombinant proteins were purified on Nickel-nitrilotriacetic acid agarose (Ni-NTA) column (Qiagen) under native conditions per manufacturer's instructions. The purified proteins were concentrated using Amicon Ultra-15 centrifugal filter (10 kd cutoff, Millipore) and reconstituted with 1×phosphate-buffered saline (PBS) to remove imidazole. The purified proteins were examined by SDS-PAGE, and the protein concentrations were determined by a Biophotometer (Eppendorf North America).

### SDS-PAGE and Western Blot analysis

SDS-PAGE was performed using 12% or 15% polyacrylamide gels [32]. Samples of rExhC were mixed with Laemmli buffer and boiled for 5 min. Gels were stained with Coomassie brilliant blue R-250. For Western Blot, samples were resolved on SDS-PAGE gel before transferred onto nitrocellulose membranes (Millipore). Membranes were probed with mouse anti-his (c-term) monoclonal antibody (Invitrogen) or rabbit polyclonal antibodies against *S. sciuri* HBXX06. The blots were subsequently incubated with HRP-conjugated goat anti-mouse IgG or HRP-labeled goat anti-rabbit IgG secondary antibodies (DingGuo Biotech). The blots were developed using the chemiluminescence blot detection reagents (Vigorous Biotech).

### Examination of rExhC activity

The *in vivo* activity of rExhC was examined by subcutaneous injection of newborn mice with 500 µg of purified rExhC or with PBS as control. Gross lesions in the skin were examined every hour post rExhC treatment. The skin tissues were collected for histological examination at the end of the experiment. The *in vitro* activity of rExhC was examined with cell cultures. BHK-21 cells were cultured in 96-well culture plates at a density of 2×10^4^ cells per well for 12 h before treatment with 15 µM rExhC. The morphological changes of treated cells were observed with a microscope.

### Flow cytometry

BHK-21 cells (2×10^5^) were cultured for 6 h and then incubated with 0, 0.3, 3 or 15 µM rExhC. Twenty-four hours after rExhC treatment, cells were harvested and stained with FITC-labeled annexin-V and propidium iodide (PI) per manufacturer's instructions (Biosea Biotech). Cells were analyzed on a FACs-Calibur flow cytometer (BD Biosciences) using the CellQuest program (BD Biosciences).

### Assessment of internucleosomal DNA fragmentation

For internucleosomal DNA fragmentation assay, DNA was extracted using TIANamp Genomic DNA blood kit (Tiangen Biotech) according to the manufacturer's instructions. In brief, at specific time points after rExhC treatment, both the floating and adherent cells were pooled. DNA was extracted from these cells and dissolved with 50 µL TE buffer (pH 8.0). These samples were electrophoretically resolved on 1% agarose gel. Fragmented DNA was visualized under ultraviolet light.

### Western Blot analysis for caspase cleavage

BHK-21 cells were cultured with 15 µM rExhC or medium only as a control for 24 h. The cells were harvested and lysed (50 mM HEPES, 150 mM NaCl, 1% Triton X-100, 5 mM EDTA, 50 mM β-glycerophosphate, 20 mM NaF, 2 mM phenylmethylsulfonyl fluoride, 10 µg/ml leupeptin and 10 µg/ml aprotinin). The cell lysates were centrifuged and the protein content was determined. Equal amounts of protein were separated by 12%SDS-PAGE. Proteins were transferred to a nitrocellulose membrane and then immunoblotted with anti-caspase-3 or anti-caspase-9 antibodies (Santa Cruz Biotechnology). The blots were subsequently incubated with HRP-conjugated goat anti-rabbit IgG secondary antibodies (DingGuo Biotech). The blots were developed using the chemiluminescence blot detection reagents (Vigorous Biotech).

### DNA release assay

DNA release was quantitatively measured using a DNA detection ELISA kit (Roche Applied Science). Briefly, BHK-21 cells were labeled with 10 µM BrdU for 24 h. The BrdU-labeled cells (1×10^4^) were cultured in 96-well plates at 37°C with 5% CO_2_ for 4 h before treatment with 15 µM rExhC or 0.1% NP-40 as a positive control for necrosis. The culture supernatants were collected at 0.5, 1, 2, 4, 8 and 16 h post treatment and examined for DNA contents using ELISA kit per manufacturer's instruction.

### Preparation of truncated rExhC

The truncated *ExhC* were amplified from pMD19-T-ExhC using the same forward primer (F: 5′-CCATGGCTATGCATTCAAAACTATTAAGTAAAT-3′) and reverse primers (T1-R: 5′-GCGGCCGCCGGAACTGTATAGCCATAGTATT or T2-R: 5′-GCGGCCGCAGCAGCTTTAATGACATCG or T3-R: 5′-GCGGCCGCATCAGCATGTCTACCTGGA or T4-R: 5′-GCGGCCGCATATGGGGAGTTTTTGATTT). The *Nco* Ι and *Not* Ι sites are underlined. These truncated rExhC were named T1-rExhC (1-228 aa), T2-rExhC (1-178 aa), T3-rExhC (1-128 aa) and T4-rExhC (1-78 aa). The construction of cloning and expression plasmids, expression and purification of truncated rExhC proteins were performed as described above.

### Functional analysis of truncated ExhC

BHK-21 cells (2×10^4^) were cultured in 96-well culture plates for 12 h before treatment with 15 µM rExhC, T1-rExhC, T2-rExhC, T3-rExhC and T4-rExhC respectively or with PBS as controls. The morphological changes of treated cells were observed with a microscope. The necrotic effects of truncated rExhC were examined using BHK-21 cells as described above.

### Establishment of B hybridoma clones

Five eight-week old female inbred BALB/c mice were immunized with recombinant ExhC-his fusion protein in emulsified Complete Freund's adjuvant at a dose of 40 µg per mouse by subcutaneous and intradermal injections (Sigma-Aldrich). Booster immunizations were performed by subcutaneous injection with rExhC in emulsified incomplete Freund's adjuvant (Sigma-Aldrich) at a dose of 40 µg per mouse. Three days after the fourth intraperitoneal injection with rExhC, splenocytes were isolated and fused with SP2/0 myeloma cells as previously described [33]. An indirect ELISA was used to screen for hybridoma clones that produced a monoclonal antibody against ExhC. Ascite fluids were obtained from the mouse peritoneal cavity [34]. Monoclonal antibodies were purified as previously described [16]. The purified antibodies were examined by SDS-PAGE and the concentrations were determined by a Biophotometer.

### Inhibition of rExhC-induced cell death by a monoclonal antibody

BHK-21 cells (5×10^4^) were seeded on 48-well cell culture plates and cultured for 12 h. Two hundred µl of rExhC (500 µg ml^−1^) was incubated with 10 µl 3E4-Ab (50 mg ml^−1^) at room temperature for 20 min before added to the cell culture. The plates were incubated at 37°C with 5% CO_2_ for 24 h before the viability of cells was determined using trypan blue dye exclusion assay [35]. An isotype IgG1 was used as a control in culturing with rExhC.

### Inhibition of rExhC-induced skin lesions by a monoclonal antibody

Groups of newborn mice (n = 9) were injected intraperitoneally with 3E4-Ab at different doses (5, 1.25, or 0.25 mg per mouse) while 1.25 mg of IgG1 control or equal volume of PBS was given as controls. One hour after antibody treatment, mice were subcutaneously injected with rExhC (500 µg per mouse). The skin lesions were observed and recorded every hour after rExhC injection. The skin lesions were scored as follows: 100% stands for a lesion area greater than 1 CM^2^; 75% for the lesion area between 0.5 and 1 CM^2^; 50% for the lesion area between 0.25 and 0.5 CM^2^; 25% for the lesion area less than 0.25 CM^2^ and 0 for no lesion. Five hours after rExhC injection, skin tissues were collected for the histopathological examination as previously described [36].

### Statistical analysis

The significance of the differences between treatment groups and controls in DNA contents, cell survivals and lesion scores was determined by the Mann-Whitney and ANOVA accordingly.

## Supporting Information

Figure S1 **rExhC induced caspase-independent cell death.** A. BHK-21 cells were cultured with 15 µM rExhC or medium only as a negative control for 1, 2, 4, 8 and 16 hours, followed by internucleosomal DNA fragmentation assay as described above. Ladder indicates DNA ladder, and Ctrl indicates control. B&C. BHK-21cells were incubated with 15 µM rExhC or medium only as a control. Twenty-four hours after rExhC treatment, the cell lysates were prepared and subjected to SDS-PAGE on 12% gel and immunoblotted with anti-caspase-3, anti-caspase-9 or anti-actin antibodies. D. BHK-21 cells were treated with vehicle or rExhC alone or pretreated with pancaspase inhibitor zVAD-fmk (50 µM) for 2 h and then incubated with rExhC (15 µM) for 8 h. Morphological changes were observed with a microscope. Arrows indicate necrotic cells.
(TIF)
