# Supplementary figures and images for: Staphylococcus sciuri Exfoliative Toxin C (ExhC) is a Necrosis-Inducer for Mammalian Cells
Source: PLoS One. 2011 Jul 29;6(7):e23145. doi: 10.1371/journal.pone.0023145 (PMC3146541; doi:10.1371/journal.pone.0023145)

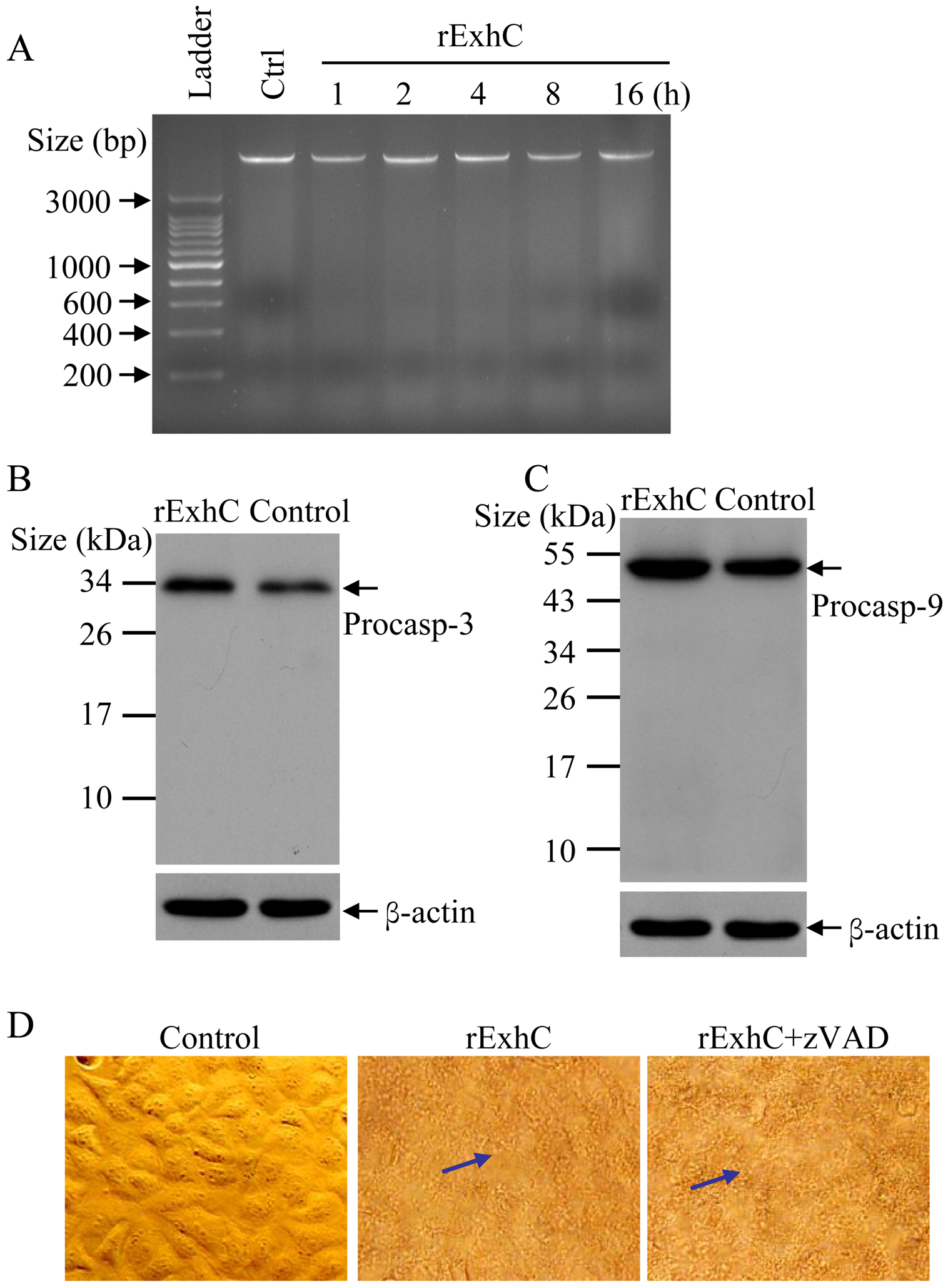

Supplement: Figure S1 — rExhC induced caspase-independent cell death. A. BHK-21 cells were cultured with 15 µM rExhC or medium only as a negative control for 1, 2, 4, 8 and 16 hours, followed by internucleosomal DNA fragmentation assay as described above. Ladder indicates DNA ladder, and Ctrl indicates control. B&C. BHK-21cells were incubated with 15 µM rExhC or medium only as a control. Twenty-four hours after rExhC treatment, the cell lysates were prepared and subjected to SDS-PAGE on 12% gel and immunoblotted with anti-caspase-3, anti-caspase-9 or anti-actin antibodies. D. BHK-21 cells were treated with vehicle or rExhC alone or pretreated with pancaspase inhibitor zVAD-fmk (50 µM) for 2 h and then incubated with rExhC (15 µM) for 8 h. Morphological changes were observed with a microscope. Arrows indicate necrotic cells. (TIF) [file pone.0023145.s001.tif]
